# PD116 Development Of A Value Framework For The Appropriate Prescription Of High-Cost Cancer Drugs In A Cancer Center

**DOI:** 10.1017/S0266462324003568

**Published:** 2025-01-07

**Authors:** Camila Quirland, Diego Carreño-Leiton, Daniela Lahoz-Quintanilla, Carlos Muñoz-Montecinos, Felipe Maza, Catalina González-Browne

## Abstract

**Introduction:**

Effectiveness, efficiency, and consistency with patient preferences are requirements for appropriate healthcare. The Complex Treatment Evaluation Committee (CTEC) at the Arturo López Pérez Foundation is a multidisciplinary committee that assesses the appropriateness of high-cost cancer drug prescriptions (HCCDP) and authorizes their use accordingly. Our study aimed to develop a value framework to assess the appropriateness of HCCDP at the Foundation.

**Methods:**

We conducted a literature review to identify appropriateness criteria for oncology prescriptions and the judgments used by the Chilean healthcare system for clinical practice guideline recommendations and reimbursement decisions for these medications. The results were discussed by the CTEC to establish a final value framework through consensus and to define a methodology to assess the appropriateness of HCCDP weekly. Annual indicators were designed to improve the agreed methods and the adequacy of prescriptions.

**Results:**

Criteria for the value framework were grouped into three categories: magnitude of clinical benefit, efficiency, and sustainability. Every criterion should be met to consider an HCCDP as appropriate. Adequacy was evaluated by assessing prescription evidence identified from electronic databases, evidence-based clinical practice guidelines, regulatory agency reports, and health technology assessment reports. From 2019 to 2022, 1,626 cases have been evaluated. Although potentially inappropriate CTEC authorizations have decreased over time, there was a growing mismatch between these decisions and the prescribing behavior of clinicians.

**Conclusions:**

By involving clinicians, managers, and health economists we developed a value framework for the timely assessment of the appropriateness of HCCDP in a hospital setting. Further research on the underlying reasons for the differences observed is needed, along with additional appropriateness criteria such as consistency with the preferences and ethical principles of patients.

